# Evaluation of a Forefront Weight Scale from an Automated Calf Milk Feeder for Holstein and Crossbred Dairy and Dairy–Beef Calves

**DOI:** 10.3390/ani13111752

**Published:** 2023-05-25

**Authors:** Kirsten T. Sharpe, Bradley J. Heins

**Affiliations:** West Central Research and Outreach Center, University of Minnesota, Morris, MN 56267, USA; sharp200@umn.edu

**Keywords:** automated milk feeder, calves, body weight, crossbreeding

## Abstract

**Simple Summary:**

The objective of this study was to evaluate the precision and accuracy of a forefront weight scale attached to an automatic calf milk feeder for Holstein and crossbred dairy calves. The forefront weight scale was compared to the gold standard electronic scale. The correlations between the forefront weight scale and electronic scale were high across all calves and all breed groups in the study. Using forefront weight scales attached to automated calf milk feeders is a reliable method to record accurate body weights of numerous breeds of calves.

**Abstract:**

Recording of body weights of dairy calves may assist producers in monitoring the health status of calves and making feed-related management decisions. Traditional methods of weighing calves can be time-consuming and labor-intensive. The objective of this study was to evaluate a forefront weight scale on stalls attached to an automated calf milk feeder system to determine the accuracy for measuring the calf body weights of Holstein and crossbred dairy calves. The study was conducted at the University of Minnesota West Central Research and Outreach Center, Morris, MN, dairy. Eighty-eight Holstein and crossbred calves were fed either 8 L/d or ad libitum milk from September 2019 to February 2020 and March 2020 to July 2020. Crossbred calves were Grazecross crossbreds composted of Jersey, Viking Red, and Normande, ProCross crossbreds composed of Holstein, Montbéliarde, and Viking Red, Limousin-sired crossbred dairy × beef bull calves, and Limousin-sired crossbred dairy × beef heifer calves. The Limousin-sired calves were from Holstein or crossbred dams. Calves were introduced to the Holm & Laue Calf Expert and Hygiene Station automatic calf feeder (Holm & Laue GmbH & Co. KG, Westerrönfeld, Germany) at 5 days of age and were weaned at 56 d. Forefront weight scales were attached to four hygiene station feeding stalls on the automated calf milk feeder, and calves were required to place both front hooves on the scale to access milk. The calf weights from the automated milk feeder were compared to the gold standard calibrated electronic scale (Avery Weigh-Tronix LLC, Fairmont, MN scale). Calves were weighed once per week using the electronic scale, and those weights were compared to the most recent weight recorded by the forefront scale. The associations of the weights from the automated milk feeder scale and the electronic scale were determined with Pearson correlations (PROC CORR of SAS) and Bland–Altman plots (PROC SGPLOT of SAS). Furthermore, PROC GLM of SAS was used to regress the electronic scale body weight on the forefront weight scale body weight for each calf. A total of 600 weight observations were used for statistical analysis. The Pearson correlation of the electronic scale compared to the forefront weight scale was high (0.991), and the concordance correlation coefficient was high (0.987). Correlations for individual calves ranged from 0.852 to 0.999 and were classified as high. Correlations of the electronic scale and forefront weight scale for breed groups ranged from 0.990 to 0.994. The slope of the regression line was 0.9153, and the 95% confidence interval was between 0.906 and 0.925. A mean bias of 0.529 kg was observed from the Bland–Altman plots. The results suggest that there is potential for the forefront weight scale to be used on automated calf milk feeders to accurately record the body weights of calves and support management decision-making, identify sick calves, and help producers determine the proper dosage of medications for calves based on body weight.

## 1. Introduction

Monitoring the body weight of dairy calves from birth may provide valuable knowledge to the producer and assist in determining diet effectiveness, management decisions, and the health status of calves [1,2]. Furthermore, many common medications for dairy calves require body weight estimation for proper dosing of the calf. It is common for body weight to be estimated by visual observation; however, this can be a variable and inaccurate method for estimating the body weights of calves. As a calf grows, visual estimation of body weight is typically overestimated [3]. Monitoring body weights early in life is a simple method that helps to ensure calves maintain growth curves necessary to support eventual breeding, calving, entrance into the lactating herd, and future production [4,5,6,7]. Numerous studies report that calves with higher ADG will become more productive cows, and changes in nutrition need to be relatively quick so as not to lose this opportunity to increase milk production of the dairy herd [4,5,6].

Electronic weight scales that are calibrated are considered to be the most accurate measure of body weight [8]. However, weight scales are not always available to the producer or may not be calibrated correctly [3]. Heart girth tapes may be used as a method for estimating body weight and have been shown to be highly correlated with body weight [1,9]. Dingwell et al. [8] reported a lower correlation between scale weight and tape weight for young calves, so there is an opportunity to increase the measurement accuracy of the weights of calves. Body weights recorded from a heart girth tape were proven to be accurate in estimating the body weight of Holstein calves, and a correlation of 0.98 was reported for the comparison of scale body weight and heart girth body weight [1]. Hoof-circumference tapes were shown to be another reliable method of estimating the birth weight of calves when compared to a spring scale [10]. However, hoof-circumference tapes were not accurate when estimating the birth weight of light and heavy calves and were determined to be accurate for calves that had body weights between 31 and 45 kg [10]. Another method of body weight estimation using hipometers was found to be inconsistent in comparison to a weight scale in Holstein calves older than 15 months and younger than 3 months [8]. Although tested in some purebred dairy breeds, the use of heart girth tapes and hoof-circumference tapes may not be accurate in estimating the weight of crossbred calves. Although methods such as the electronic weight scale [11] and estimated body weight from heart girth measurements have been shown to be effective, these methods require additional labor, time, and physical energy to collect the body weights [12]. Furthermore, surveys have shown that dairy producers find weighing calves with a weight scale to be time-consuming and costly [12].

Automated milk feeding systems for dairy calves have been growing in popularity in the United States [2,13]. Automated milk feeders may provide producers with insight into calf health, nutrition, growth, and behavior [14]. Some automated calf milk feeding systems on farms have forefront weight scales attached to the feeder or attached to feeding stalls to record calf weights every time a calf comes to the milk feeder for a milk allowance [15]. Recently, Cantor et al. [16] reported a correlation of greater than 0.90 for a partial-weight scale attached to a DeLaval Combi automated calf feeder for 20 Holstein heifer calves.

Forefront weight scales are an option that producers may install on some automated milk feeders for an additional cost. Each time a calf visits the feeder, the calf milk feeder software automatically records and processes calf weights and stores the data in the system. This may reduce labor involved with weighing calves manually and may assist in identifying health challenges or diseases in calves. Research studies that investigate the accuracy of forefront weight scales attached to automated milk feeders are limited. Furthermore, no studies have compared the weights of crossbred dairy and beef x dairy calves with an electronic weight scale versus a forefront weight scale attached to an automated calf milk feeder. Therefore, the objective of this study was to evaluate the forefront weight scale attached to an automated calf milk feeder by comparing it to a gold-standard electronic scale. Furthermore, the accuracy of the forefront weight scale in measuring body weights was determined for purebred Holstein calves and crossbred dairy calves and crossbred beef x dairy calves.

## 2. Materials and Methods

The study was conducted at the University of Minnesota West Central Research and Outreach Center (WCROC; Morris, MN). All the animal procedures involving animal care and management were approved by the University of Minnesota Institutional Animal Care and Use Committee. The WCROC research dairy herd is a 275-head, low-input, and grazing dairy herd. Since 2000, a crossbreeding approach has been used in the herd to produce cows optimized for grazing systems. For the dairy herd, cows are calved during spring (March to May) and autumn (September to November). The WCROC herd initiated a crossbreeding program in 2000 and has a 3-breed rotational crossbreeding system of the Montbeliarde, Viking Red, and Holstein breeds and another 3-breed rotational crossbreeding system of the Normande, Jersey, and Viking Red breeds. The calves in this study were Holstein (1964 genetic control Holstein [17] or contemporary purebred Holstein) and either a crossbred composed of Viking Red, Montbéliarde, and Holstein or a crossbred composed of Jersey, Normande, and Viking Red [18,19,20,21]. Beginning in 2018, Limousin beef sires were used in the herd as mates for Holstein and crossbred cows. Therefore, the Limousin-sired calves were all crossbred calves. More details on the breed composition of calves are discussed in Pereira et al. [22,23].

Data were collected for a total of 88 dairy and beef x dairy calves born during 2 calving seasons (1 autumn season and 1 spring season): 47 calves were born from September to December 2019, and 41 calves were born from March 2020 to May 2020. Across the 2 study seasons, breed groups of calves were 1964 Holstein (n = 3), purebred contemporary Holstein (n = 13), Grazecross crossbreds including combinations of Jersey, Viking Red, and Normande (n = 6), ProCross crossbreds including combinations of Holstein, Montbéliarde, and Viking Red (n = 22), beef x dairy Limousin crossbred bull (LimoB; n = 22), and beef × dairy Limousin crossbred heifer (LimoH; n = 22).

Calves were separated from their dams at birth and raised indoors in individual pens until 5 days of age. While in the individual pens, calves were fed 1.9 L of colostrum per 41 kg of body weight twice on day one and then transitioned to milk twice daily beginning on day 2 until day 4. On day 5, calves were moved to the automated calf milk feeder pens. Two pens separated calves into two groups: 24 in the first pen (oldest calves) and 23 in the second pen (youngest) during the first calving season. During the second calving season, the first pen contained 21 calves, and the second pen contained 20 calves. Calves were fed on the automated calf milk feeder from 5 days of age until 56 days.

All calves were provided whole milk from a Holm & Laue Calf Expert and Hygiene Station automatic calf feeder (Holm & Laue GmbH & Co. KG, Westerrönfeld, Germany). Whole milk was fed at 13% of the total solids of pasteurized saleable and non-saleable milk. The milk averaged 4.2% fat, 3.3% protein, and 5.5% other solids. Ad libitum milk was offered to 44 of the 88 total calves in this study from the automated calf milk feeder. The other 44 calves were offered 8 L of milk per day from the automated calf milk feeder. The two treatments were spread evenly through the breed groups during both seasons. After the calves were moved to the automated calf milk feeder pen, human assistance was provided by moving the calf to the nipple in the drinking stall until the calf was observed consuming milk independently. Free choice texturized calf starter and water was provided when calves were moved to the automated calf milk feeder pens. Calf starter was 19% crude protein from corn, oats, soybean meal, soybean oil, and minerals.

Across both seasons of the study, breed groups of calves were Holstein (9 calves for ad libitum and 7 calves for 8-L/day), ProCross crossbreds (10 calves for ad libitum and 12 calves for 8-L/day), Grazecross crossbreds (3 calves for ad libitum and 3 calves for 8-L/day), and Limousin-sired crosses from ProCross and Grazecross cows (LH heifer calves (12 calves for ad libitum and 10 calves for 8-L/d); LB bull calves (10 calves for ad libitum and 12 calves for 8-L/day). The unbalanced numbers of calves per breed group were because of the sex ratios of Holstein and crossbred dairy calves, and the dairy herd is two-thirds crossbreed (mostly ProCross crossbreds) and one-third Holstein. Furthermore, Limousin was used for mating 40% of the dairy herd. Calf sex is unknown until birth, and calves that were used survived past 3 d of age for the study, so it can be very difficult to predict sex ratios by breed groups of calves before a study begins. The breed groups varied in the number of calves; however, these calves were spread across 2 calving seasons, so they contributed meaningful information for breed group comparisons.

The automated calf milk feeder was equipped with 4 feeding stalls, each of which had an attached forefront weight scale. Each time a calf visited the feeder, a radio frequency identification system attached to the stall read the calf ID to determine the milk allotment to the calf. To reach the nipple to access milk, the calf was required to place both front feet on the scale. The automated calf milk feeder recorded the weight from the forefront weight scale and calculated the full body weight of a calf each time the calf visited the feeder. The manufacturer used algorithms and raw data to determine the weight of the calves on the forefront scale. Algorithms are proprietary and are not provided to farmers or researchers. To compare the accuracy of the forefront scale, each calf was weighed once per week at 1300 h on an electronic scale (Avery Weigh-Tronix LLC, Fairmont, MN scale head). The most recent recorded weight from the specific weight data for the automated calf milk feeder was recorded and compared to the electronic scale. Both the forefront scale and electronic scale were calibrated by a weight scale calibration company prior to the study.

Pearson correlations were used to evaluate the agreement between the electronic scale and the forefront weight scale via PROC CORR of SAS 9.4 [24]. Furthermore, PROC GLM of SAS 9.4 was used to regress the electronic scale body weight on the forefront weight scale body weight for each calf to estimate a regression coefficient and 95% confidence intervals for all calves. Bias correction factors, concordance correlation coefficient (CCC; [25]), location shift, and scale shift for the electronic scale and automated calf milk feeder scale were determined using SAS software.

Bias correction factors measured how far the regression line strayed from the ideal line and ranged from 0 to 1, with 1 indicating full agreement with the ideal line [26,27]. The CCC (Pearson correlation coefficient × bias correction factors) provided an evaluation of the reproducibility of the measurements taken from the forefront weight scale in comparison to the gold standard electronic scale measurements. The CCC ranged from −1 to 1, with perfect agreement at 1 [25]. Pearson correlations, linear coefficient of determination, CCC, and bias correction factors were classified according to Hinkle et al. [28], with 0.0 to 0.3 as negligible, 0.3 to 0.5 low, 0.5 to 0.7 moderate, 0.7 to 0.9 high, and 0.9 to 1.0 very high. The location shift determined values that were under or over-predicted. A negative value indicated over prediction, and a positive value indicated under prediction. A value of 0.0 represented a perfect agreement between the two weight scales. The difference in standard deviation between the electronic scale and the automated calf milk feeder scale was indicated by the scale shift. A scale shift of 1.0 indicated perfect agreement between the electronic scale weight and the forefront scale weight [26].

Mean difference and 95% confidence intervals for the electronic scale weight and the automated calf milk feeder forefront scale were determined with Bland–Altman plots using PROC SGPLOT of SAS 9.4 [24]. The limits of agreement were ±1.96 standard deviations from the bias [29], and statistical significance was declared at *p* < 0.01.

## 3. Results

Across the two study seasons, three calves (two from the autumn of 2019 and one from the spring of 2020) were removed. On each weigh day, the three calves had consistently and drastically lower weights recorded by the automated calf milk feeder compared to the electronic scale. The Pearson correlation coefficients of the calves that were removed were 0.957, 0.887, and 0.953. The three calves had a mean weight of 78.5 kg with a standard deviation of 26.3 kg from the electronic scale and a mean weight of 23.6 kg and a standard deviation of 11.4 kg from the forefront weight scale. However, it is uncertain why these three calves had inaccurate weights, but it could be related to how the calf placed its feet on the forefront weight scale while feeding.

During the autumn of 2019, the maximum weight was 121.1 kg for the electronic scale and 132.9 kg for the forefront weight scale. During the spring of 2020, the maximum weight was 141.9 kg for the electronic scale and 145.6 kg for the forefront weight scale

Results for the electronic scale weights, forefront scale weights, and the correlation of the electronic scale and forefront scale weights across breeds and treatments are shown in Table 1. A total of 600 observations between the electronic scale weight and the automated calf milk feeder scale were recorded across the two seasons. The mean electronic scale weight across all calves was 69.9 kg ± 18.2 kg, and the mean automated calf milk feeder scale weight across all calves was 70.5 kg ± 19.8 kg. The Grazecross breed group had calves that had the lowest body weight with the electronic scale (61.7 kg ± 17.7 kg) and the forefront weight scale (61.7 kg ± 19.4 kg). The heaviest calves for body weight were the Limousin crossbred bull calves, with 73.1 kg ± 18.8 kg from the electronic weight scale and 73.8 kg ± 20.1 kg from the forefront weight scale. The correlation for all breed groups of calves was high (>0.991, *p* < 0.001) for the electronic scale and forefront weight scale. The breed groups ranged from 0.990 to 0.994 (*p* < 0.001) for correlation for the electronic scale and forefront weight scale. Furthermore, the correlation was high for calves fed ad libitum (0.990) and for calves fed 8 L per day (0.993) for the electronic scale and forefront weight scale.

Table 2 shows regression coefficients and 95% confidence intervals of calf weights estimated using a forefront weight scale attached to an automated milk feeder and weights recorded with an electronic scale for all calves and breed groups of calves. The precision of the forefront scale was very high (0.92) across all breeds, with each individual breed type having high precision as well (>0.90). Regression coefficients ranged from 0.90 for 1964 Holstein and ProCross heifer calves to 0.93 for Limousin crossbred-sired heifer calves. Individual calves ranged from 0.790 to 1.15 for regression coefficient estimates. One Holstein heifer calf had a regression coefficient of 0.50 but had a correlation of 0.85 between the two weight scales.

The bias correction factor, CCC, location shift, scale shift, mean differences from Bland–Altman Plots, and lower and upper 95% limits of agreement are shown in Table 3. The bias correction factor (0.996) and CCC (0.987) across all calves and breed groups were high and indicated substantial precision of the forefront weight scale and high agreement between the gold-standard electronic scale and the forefront weight scale.

**Figure A1:**
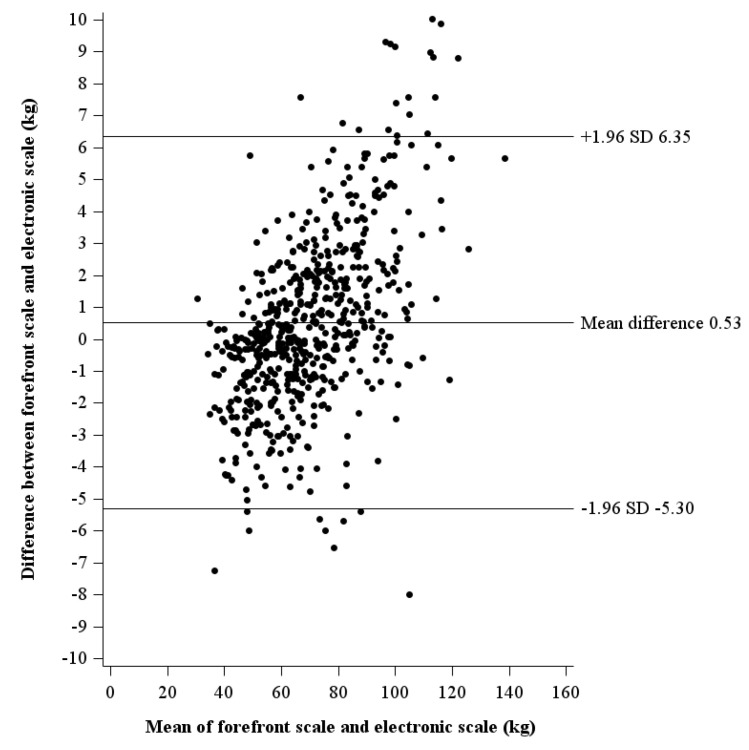
Agreement between the electronic weight scale and the forefront weight scale for all calves (n = 88). The middle line indicates the zero line, and outer lines indicate the lower and upper 95% CI.

**Figure A2:**
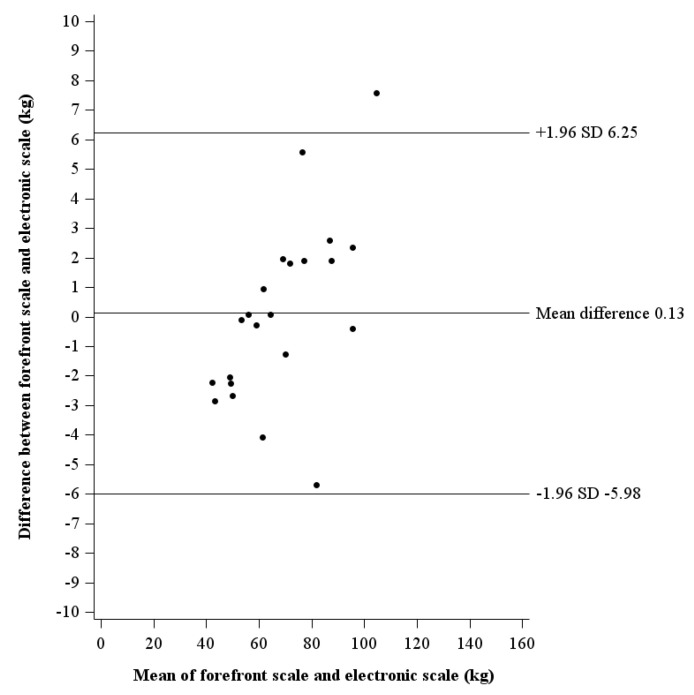
Agreement between the electronic weight scale and the forefront weight scale for 1964 Holstein heifer calves (n = 3). The middle line indicates the zero line, and outer lines indicate the lower and upper 95% CI.

**Figure A3:**
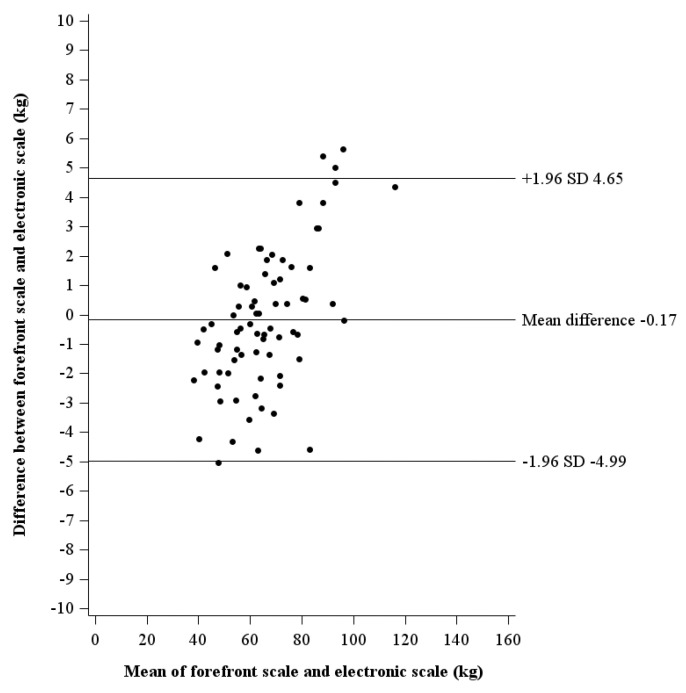
Agreement between the electronic weight scale and the forefront weight scale for Holstein heifer calves (n = 13). The middle line indicates the zero line, and outer lines indicate the lower and upper 95% CI.

**Figure A4:**
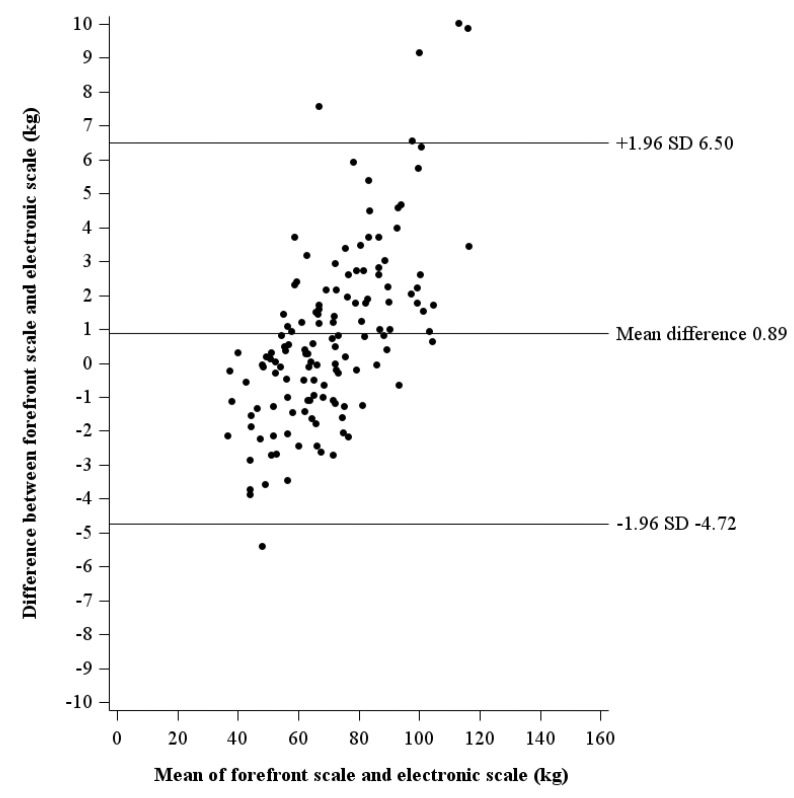
Agreement between the electronic weight scale and the forefront weight scale for ProCross heifer calves (n = 22). The middle line indicates the zero line, and outer lines indicate the lower and upper 95% CI.

**Figure A5:**
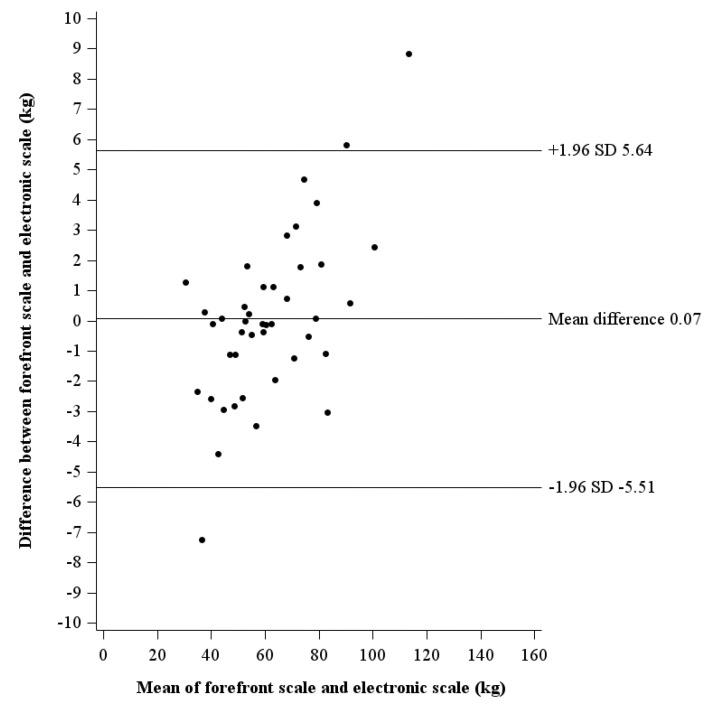
Agreement between the electronic weight scale and the forefront weight scale for Grazecross heifer calves (n = 6). The middle line indicates the zero line, and outer lines indicate the lower and upper 95% CI.

**Figure A6:**
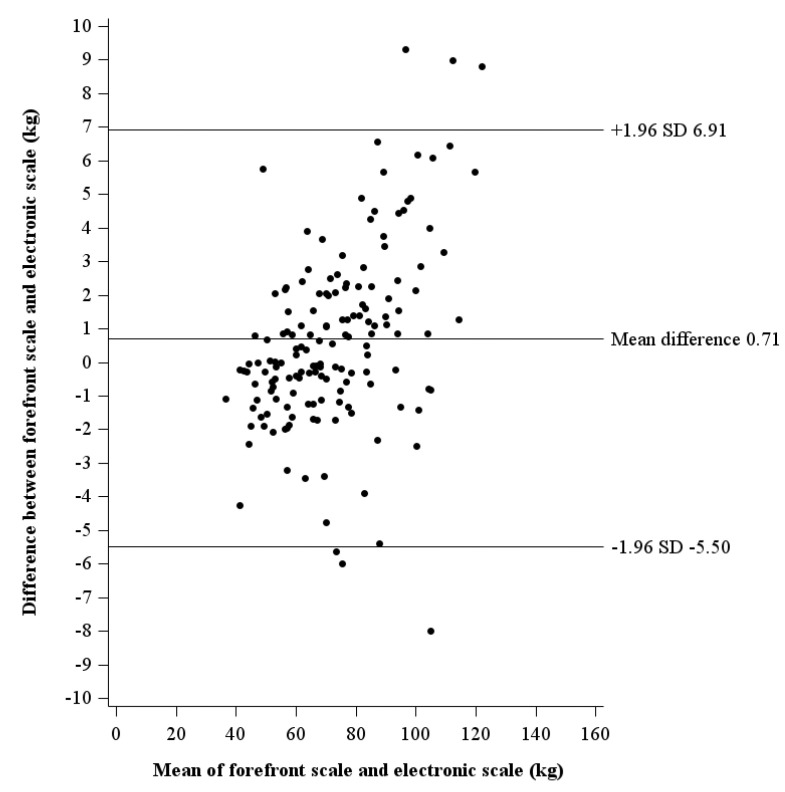
Agreement between the electronic weight scale and the forefront weight scale for Limousin-sired crossbred bull calves (n = 22). The middle line indicates the zero line, and outer lines indicate the lower and upper 95% CI.

**Figure A7:**
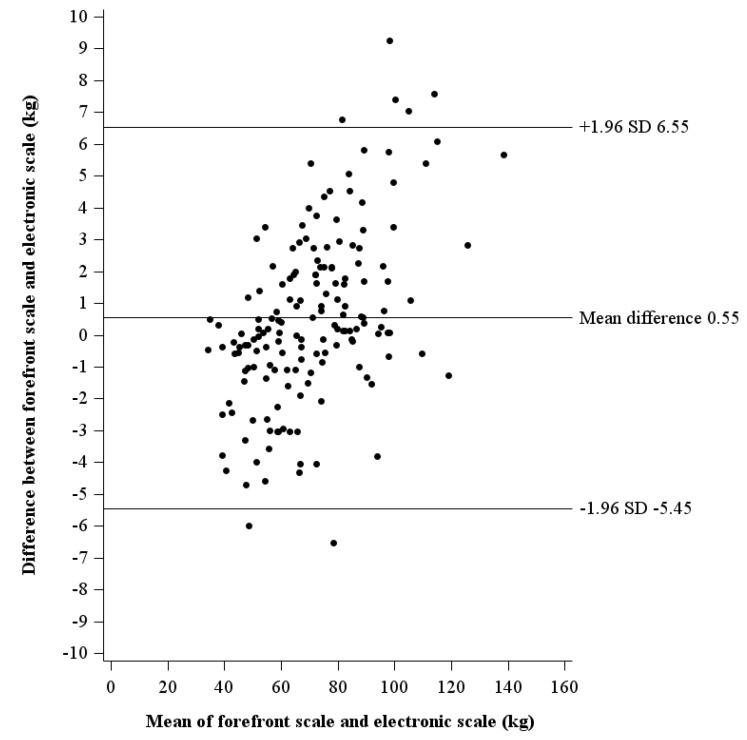
Agreement between the electronic weight scale and the forefront weight scale for Limousin-sired crossbred heifer calves (n = 22). The middle line indicates the zero line, and outer lines indicate the lower and upper 95% CI.

Appendix Figure A1, Figure A2, Figure A3, Figure A4, Figure A5, Figure A6 and Figure A7 show Bland–Altman plots for all calves, 1964 Holstein, Holstein, ProCross, and Grazecross heifer calves, as well as Limousin-sired crossbred bull calves and Limousin-sired crossbred heifer calves. For all Bland–Altman plots, most of the observations were near the zero line, and few outliers occurred outside of the 95% CI. The lower and upper 95% confidence interval of the Bland–Altman plots included most of the calf body weights. The Bland–Altman plot (Appendix A Figure A1) had a mean difference of 0.529 ± 2.91 kg, and the limits of agreement were from −5.30 to 6.35 for all calves.

## 4. Discussion

The results indicate that all breed groups of calves were measured accurately, and farms with breeds other than Holstein may record accurate weights from an automated calf milk feeder forefront weight scale. Quite possibly, the gains experienced by ad libitum calves were not as recognized by the automated calf milk feeder software algorithm compared to a “typical” feeding regime because of the numerically lower correlation and higher standard deviation of weights of the ad libitum calves compared to the 8 L calves. Cantor et al. [16] found the correlation between a partial weight scale attached to an automated milk feeder to be high (>0.90) across all Holstein calves in that study. Furthermore, that study reported regression coefficients ranging from 0.88 to 1.12 for Holstein heifer calves which are similar to the current study. However, the forefront weight scales for Cantor et al. [16] and the current study were from different manufacturers and different calibration algorithms to determine the weight of a calf. The results from the current study for high correlations and regression coefficients indicate the high precision of the weights between the electronic scale and the forefront weight scale.

Furthermore, low location shift values across all calves and breed groups indicated high reproducibility by the forefront weight scale. Interestingly, purebred Holstein heifer calves had a numerically lower correlation (0.992) compared to the Grazecross (0.993) and ProCross (0.994) calves (Table 1) but equal CCC when compared to ProCross crossbred calves (0.988). Furthermore, the CCC of the 1964 Holsteins (0.986) indicated the least correspondence between the two scales across all the breeds, and the scale shift of 1964 Holsteins was furthest from perfect agreement across the breeds (1.106). However, this breed group had three calves and 1964 genetic Holsteins are found only in one research herd in the US. Removal of these three calves may have decreased variation in the weights of this study. However, the correlation between the electronic scale and forefront scale for the 1964 Holsteins has the same value as all calves and the Limousin-sired crossbred dairy × beef heifer calves. The bias correction, CCC, mean difference, and confidence intervals were similar for the 1964 Holsteins and the other breeds groups. The 1964 Holstein calves provided meaningful comparisons to other breed groups, even with the small number of observations.

For the ProCross heifer calves and Limousin-sired crossbred dairy × beef heifer calves (Appendix Figure A4, Appendix Figure A7), more observations appeared outside of the 95% CI, but this was expected because these breed groups had the highest location shift and the ProCross calves had the highest mean difference for the electronic weight scale and forefront weight scale. For the Bland–Altman plots for 1964 Holsteins, Holsteins, Grazecross crossbreds, and Limousin-sired crossbred dairy × beef bull calves, few outliers fell outside of the 95% CI.

The forefront weight scale from the Holm & Laue Calf Expert recorded calf body weight with high precision and accuracy for calves to 8 weeks of age and should be used as a benchmark for calf weights. Recording calf weight by hand or with a calf weight tape requires additional labor, may be subject to bias, and may not be very accurate when weighing many calves. Furthermore, farms with a large number of calves do not have the labor force to record numerous weights with high accuracy to monitor the health of dairy calves on milk. Forefront calf weight scales are an additional cost when purchasing an automated calf milk feeder. However, if a farm purchases an automated calf milk feeder, the forefront weight scales should be added to the automated calf milk feeder when purchased to add additional monitoring information to the calf-raising enterprise. A forefront weight scale records body weight daily and at every feeding, so farms may have advantages in health with the added precision technology of an automated calf milk feeder.

The current study compared an electronic weight scale and forefront weight scale from an automated calf milk feeder for calves weighed only once per week and found high precision and accuracy in the forefront weight scales for calves to 56 d of age. Quite possibly, calves may have been weighed more frequently throughout the study (twice per week) to increase the number of observations and, quite possibly, the accuracy of the weights.

The correlation of weights from the forefront scale and electronic scale may have been affected by gut fill from calves that had just ingested milk. Calves are allowed to consume milk any time of day, and their weight is affected by gut fill [30,31]. Furthermore, calves were allowed to drink water ad libitum, and water intake may have been affected by gut fill from water intake as well [30]. The weights from the forefront scale may have been influenced by the way the calves stand and the weight distribution as the calves stand. Possibly, the three calves that were deleted in the study had a different weight distribution when standing on the forefront scale, which caused their weight to be significantly different from the electronic scale. However, Chapinal et al. [32] reported that weight distribution in dairy cows was not affected by the elevation of the front legs. Additional research should evaluate the forefront scales for calves of different weights for algorithm improvement. Further research should be conducted on forefront weight scales on an automated calf milk feeder for calves that are weaned later than 56 d and possibly up to 112 d of age (4 months) because some farms are weaning calves at greater than 56 d of age. The forefront weight scales are useful for farms to estimate body weight from calves fed on an automated calf milk feeder.

Based on the high reproducibility of the forefront scale, an electronic weight scale may no longer be needed in calf systems that utilize an automated calf milk feeder with an attached forefront weight scale. The use of a forefront scale on an automated calf milk feeder may reduce labor associated with fetching calves for weight measurements. Furthermore, an automated calf milk feeder weight scale records the weights of calves each time they visit the automated calf milk feeder, and multiple weights may be recorded from each calf every day, and these weights may provide insight into calf health and the effectiveness of a calf feeding program.

## 5. Conclusions

The correlation, precision, and accuracy were high for the forefront scale attached to an automated calf milk feeder scale for purebred Holstein and crossbred dairy and crossbred beef x dairy calves. Correlations of the electronic scale and forefront weight scale for breed groups ranged from 0.990 to 0.994. The mean bias was low for the comparison of the two weight systems. Based on the high correlation, precision, and accuracy, the results of this study suggest that the automated calf milk feed scales, rather than a separate weigh scale, may be used as a benchmark for pre-weaned dairy calves.

## Figures and Tables

**Table 1:** Number of observations, breed groups, mean body weights from an electronic scale and a forefront weight scale attached to an automated milk feeding system and correlations for all calves, breed groups of calves, and by milk allowance.

Observations (N)	Animals (N)	Breed Group	Electronic Scale Weight (kg)	SD	Forefront Scale Weight (kg)	SD	Correlation	*p*-Value
600	88	All calves	69.9	18.2	70.5	19.8	0.991	0.001
22	3	1964 Holstein	68.4	17.1	68.5	18.9	0.991	0.001
75	13	Holstein	65.7	15.0	65.5	16.4	0.992	0.001
43	6	Grazecross crossbred ^1^	61.7	17.7	61.7	19.4	0.993	0.001
136	22	ProCross crossbred ^2^	70.5	17.7	71.4	19.5	0.994	0.001
159	22	Limousin bull crossbred ^3^	73.1	18.8	73.8	20.1	0.990	0.001
165	22	Limousin heifer crossbred ^3^	70.8	19.0	71.3	20.3	0.991	0.001
292	44	Ad libitum ^4^	73.7	20.0	74.1	21.8	0.990	0.001
308	44	8 L ^5^	66.4	15.6	67.0	17.0	0.993	0.001

^1^ Grazecross = 3-breed rotational crossbred calves of the Normande, Jersey, and Viking Red breeds. ^2^ ProCross= 3-breed rotational crossbred calves of the Montbéliarde, Viking Red, and Holstein breeds. ^3^ Limousin-sired heifer and bull calves from Holstein, Grazecross, and ProCross dams. ^4^ Ad libitum milk was offered to 44 calves in the study. ^5^ 8 L of milk per day were offered to 44 calves in the study.

**Table 2:** Regression coefficients and 95% Confidence intervals of calf weights estimated using a forefront weight scale attached to an automated milk feeder, and weights recorded with an electronic scale for all calves and breed groups of calves.

Calf Type	Estimate	SE	95% CI
All calves	0.915	0.005	0.906–0.925
1964 Holstein	0.896	0.027	0.838–0.953
Holstein	0.908	0.013	0.881–0.935
Grazecross crossbred ^1^	0.905	0.017	0.871–0.939
ProCross crossbred ^2^	0.898	0.009	0.881–0.915
Limousin bull crossbred ^3^	0.923	0.011	0.902–0.944
Limousin heifer crossbred ^3^	0.928	0.010	0.908–0.948

^1^ Grazecross = 3-breed rotational crossbred calves of the Normande, Jersey, and Viking Red breeds. ^2^ ProCross= 3-breed rotational crossbred calves of the Montbéliarde, Viking Red, and Holstein breeds. ^3^ Limousin-sired heifer and bull calves from Holstein, Grazecross, and ProCross dams.

**Table 3:** Bias correction factor, concordance correlation coefficient (CCC), location shift, scale shift, The Bland–Altman mean difference, and upper and lower 95% limits of agreement for an electronic scale compared with a forefront weight scale attached to an automated calf milk feeder.

Breed Group	Bias Correction	CCC	Location Shift	Scale Shift	Mean Difference	Mean Difference SD	Lower 95% CI	Upper 95% CI
All calves	0.996	0.987	0.028	1.083	−0.529	2.91	−6.24	5.18
1964 Holstein	0.995	0.986	0.008	1.106	−0.133	3.06	−6.12	5.86
Holstein	0.996	0.988	−0.011	1.092	0.168	2.41	−4.56	4.89
Grazecross crossbred ^1^	0.996	0.989	0.004	1.098	−0.069	2.79	−5.54	5.40
ProCross crossbred ^2^	0.994	0.988	0.048	1.007	−0.886	2.81	−6.38	4.61
Limousin bull crossbred ^3^	0.997	0.987	0.037	1.073	−0.709	3.10	−6.79	5.37
Limousin heifer crossbred ^3^	0.997	0.988	0.028	1.067	−0.550	2.99	−6.43	5.33

^1^ Grazecross = 3-breed rotational crossbred calves of the Normande, Jersey, and Viking Red breeds. ^2^ ProCross= 3-breed rotational crossbred calves of the Montbéliarde, Viking Red, and Holstein breeds. ^3^ Limousin-sired heifer and bull calves from Holstein, Grazecross, and ProCross dams.

## Data Availability

Data generated from this experiment are available on request from corresponding authors.
